# Optimising plant growth, biomass partitioning, and nitrogen use efficiency in taro (*Colocasia esculenta* (L.) Schott)

**DOI:** 10.3389/fpls.2025.1731490

**Published:** 2026-01-29

**Authors:** Laura Steel, Diogenes L. Antille, Roslyn M. Gleadow

**Affiliations:** 1 School of Biological Sciences, Monash University, Melbourne, VIC, Australia; 2 Department of Ecological, Plant and Animal Sciences, La Trobe Institute for Sustainable Agriculture and Food, School of Agriculture, Biomedicine and Environment, La Trobe University, Bundoora, VIC, Australia; 3 CSIRO Agriculture and Food, Canberra, ACT, Australia; 4 Engineering Department, Harper Adams University, Newport, Shropshire, United Kingdom; 5 Institute for Climate, Energy & Disaster Solutions, Australian National University, Canberra, ACT, Australia

**Keywords:** aroid lilies, calcium oxalate, climate change adaptation, corm, food security, Pacific Islands, raphides, resource allocation

## Abstract

**Introduction:**

Taro (*Colocasia esculenta*) is the fourth most important root crop globally, yet it remains understudied. Productivity is frequently constrained by nutrient-depleted soils. This study investigates how varying nitrogen (N) levels affect taro growth, particularly biomass accumulation, sucker production, and the formation of calcium oxalate raphides, which can be harmful when ingested. We hypothesized that: (1) Growth and photosynthetic rate are highest in plants receiving the highest concentration of nitrogen; (2) Optimal corm development occurs when N is neither deficient nor excessive; (3) Sucker production increases when corm N needs are met; (4) Tissue calcium concentration (a proxy for calcium oxalate) rises when growth is limited by N.

**Methods:**

Taro plants were grown using nutrient solutions with N concentrations ranging from 2.5 to 20 mM N. Plants were harvested at different growth stages up to 10 months to capture corm formation, filling, maturity and post-maturity stages. Biomass and nutrient concentrations were measured and nitrogen use efficiency indices were calculated.

**Results:**

The highest overall biomass was at 15 mM N, but corm biomass was highest in plants grown at the 5 and 10 mM N treatments. Sucker number and biomass increased with N concentration. Calcium concentration showed a strong positive correlation with N in corms but a weak negative correlation in leaves.

**Discussion/conclusion:**

Hypotheses 2 and 3 were supported; Hypothesis 1 was not. The optimal N level for maximizing corm biomass without compromising nutritional quality is around 10 mM N. These findings will inform biophysical models for taro to help its development as a food and nutrition security crop.

## 1 Introduction

Crops with underground storage organs are the second most important source of carbohydrates globally, after cereals (Villordon et al., 2014; Gregory and Wojciechowski, 2020; Zierer et al., 2020). One such crop is taro (*Colocasia esculenta* L., Schott), a tropical, perennial plant eaten by over 200 million people in the Indo-Pacific region (Lebot, 2009; Kaushal et al., 2015). Taro is an aroid lily (Araceae) with a swollen underground stem known as a corm. All parts of the plant are edible except for the peel of the corm and the roots (Mergedus et al., 2015). Taro is a major staple in the Pacific Islands where it has been cultivated for many thousands of years and holds great cultural significance (Bradbury and Holloway, 1988; Kinaston et al., 2014). Across the Pacific region, food production has declined by 5-37% per capita this century due to factors associated with climate change, such as; increasingly erratic or less reliable rainfall (CSIRO et al., 2015), outbreaks of pests and diseases (Singh et al., 2012), rising sea levels (Myrans et al., 2024), and declining soil fertility (Antille et al., 2022; Susumu et al., 2023). Other contributing factors are a decline in the farm labor force and lack of mechanization in some systems (Meier et al., 2023). Reversing this decline in food and nutrition security in the region is intimately interwoven with taro productivity, yet research examining ways to increase taro production and quality is limited (Lebot et al., 2018).

Taro has the lowest field productivity of all root and tuber crops in Oceania, producing 6–7 tons fresh weight per hectare compared to 10–12 tons for other storage organ crops, such as cassava and sweet potato (FAOSTAT, 2023). Studies in Samoa and Fiji have reported significant yield gaps in taro, as determined by the difference between actual and attainable corm yields under current and best management practices, respectively (Antille et al., 2023; Beletse et al., 2025). While the area harvested globally has doubled this century (from approximately one to two million ha), the area under taro cultivation in Oceania has remained at around 50,000 ha (FAOSTAT, 2023). Recent reviews have identified that taro remains an under researched, ‘orphan crop’ (Aditika et al., 2022; Lebot and Ivančič, 2022; Ferdaus et al., 2023), with a need to explore the optimization of micropropagation conditions and biotechnology (Deo et al., 2009; Lebot et al., 2018; Manju et al., 2023).

Crop models can be used to predict how variables such as climate, soil, water, and nutrients influence yield and inform pathways for crop improvement (Keating et al., 2003). Beletse et al. (2025) expanded the capability of biophysical models such as APSIM (https://www.apsim.info/) by incorporating the APSIM-Taro module (Crimp et al., 2017) into the APSIM Next Gen framework (Holzworth et al., 2018) for select taro varieties. Taro is considered to prefer fertile soil that is high in nitrogen (Bradbury and Holloway, 1988; Manrique, 1994). However, data concerning the impact of fertilizers on taro productivity, growth rate, time to maturity, nutrient recovery, and corm biomass is lacking (Deenik et al., 2013; Matthews and Ghanem, 2021; Antille et al., 2023; Ferdaus et al., 2023). Traditional taro farmers rarely use fertilizers or organic amendments and broad recommendations are largely unavailable, with some exceptions (e.g (Moles et al., 1984; Deenik et al., 2013; Moa, 2018). Official recommendations for nutrient management in Pacific taro-based systems may not be reliable, as they do not reflect recent developments in plant breeding (Antille et al., 2022).

In other crops with underground storage organs, increasing N supply can result in the allocation of biomass away from the storage organs and towards the shoots (Gregory and Wojciechowski, 2020; Zierer et al., 2020). Therefore, increasing N supply alone may not increase the production of corm biomass in taro. From an agronomic perspective, a convenient way to determine the ‘optimum’ N application rate is to identify the point at which the ratio of corm biomass to nitrogen applied is maximized (nitrogen use efficiency). Two useful indices of nitrogen use efficiency (NUE) are the apparent fertilizer N recovery (AFNR) and the partial factor productivity of applied N (PFP_N_). Both indicators can be directly derived from the data collected in controlled environments or the field in a relatively straightforward manner (Antille and Moody, 2021). To determine the optimum rate of N fertilizer and calculate NUE, taro plants were grown under controlled environmental conditions using a complete fertilizer containing one of five different nitrogen concentrations (2.5, 5, 10, 15 and 20 mM N). N was supplied as nitrate and ammonium (5:1, mole:mole). Plants were harvested at regular intervals for 10 months to capture corm formation (Harvest 1-2), corm filling (Harvests 2-3), maturity (Harvest 3) and post-maturity (Harvest 4). All other nutrients were held constant and were not limiting for growth. To quantify the downstream effects of nitrogen supply on the composition of taro leaves and corms, a range of macro- and micronutrients were measured alongside total N and the C:N ratio (as an indicator of sink strength).

Like all Aroid lilies, taro plants are ‘acrid’, containing raphides of calcium oxalate (CaOx), which must be removed through processing before consumption (Bradbury and Holloway, 1988; Temesgen and Retta, 2015). Reducing calcium oxalate content is an important consideration for taro breeders (Kristl et al., 2021) that may also be affected by altered fertilizer regimes. To assess whether changes in N supply would result in a change in acridity, calcium concentrations of leaves and corms were determined as a proxy for calcium oxalate (Lloyd et al., 2021).

It was hypothesised that:

1. Growth and photosynthetic rate would be highest in plants receiving the highest concentration of nitrogen.
2. Corm biomass would be greatest in plants supplied with 5–15 mM N, as optimal corm development occurs when N is neither deficient nor excessive.
3. Sucker production would increase with increasing N supply, once the corm N requirement was met.
4. Calcium concentration of leaves and tubers would be higher (as an estimate of acridity) where N supply limited growth.

## 2 Materials and methods

### 2.1 Plant material and growing conditions

Taro plants were grown following Lloyd et al. (2021). Bare-rooted plantlets (~20 cm tall) of 110 two-week-old taro plants (*cv*. Bun Long, an eddoe variety) were purchased from Tropical Exotics Wholesale Nursery, Ningi (Queensland, Australia) and transplanted into individual pots (diameter = 250 mm, capacity = 8 L) containing Debco Seed & Cutting Premium Germinating Mix (Evergreen Garden Care Australia). To ensure establishment, plantlets were watered to saturation daily and provided with liquid fertilizer once per week for seven weeks (All-Purpose Thrive^®^, N:P:K ratios of 25:5:8.8). After this, plants were randomly allocated to one of five treatment groups, each receiving a modified Hoagland’s Solution, differing only in nitrogen concentration (2.5, 5, 10, 15, and 20 mM N). The doses of nitrogen were chosen to extend from deficiency to excess nitrogen (Manrique, 1994; Reuter and Robinson, 1997). Nitrogen was supplied as nitrate and ammonium, in a 5:1 mole:mole ratio with sodium as the balancing cation, following Gleadow et al. (1998). Ammonia was included because it is common source of N, particularly in swampy conditions where taro is often grown (Deenik et al., 2013). Other nutrient concentrations remained constant and non-limiting for growth (Gleadow et al., 1998): 6.2 mM KCl, 6.2 mM CaCl_2_, 0.25 mM MgSO_4_, 0.125 mM KH_2_PO_4_, 4 µM FeEDTA; Micronutrients were 20 µM H_3_BO_3_, 46 µM MnSO_4_, 15 µM CuSO_4_, 46 µM ZnSO_4_.7H_2_0 and 26.5 µM NH_4_Mo_7_O_4_.4H_2_O.

The overall N:P ratio (mole:mole) increased as follows with increasing nitrogen concentration: 1.5, 8.5, 17, 25.5 and 34 (**Supplementary Table S1**). Each plant received 1 L of nutrient solution twice per week. Pots were flushed once per week with 1 L of fresh water to prevent the accumulation of salts. Given that taro prefers moist or even wet soil conditions, plants were also watered using a sprinkler for one minute each day (~500 mL). Plants received a combined total of ~6.5 L of water and nutrient solution each week. The pH of the nutrient solution before application to the pots was 3.6 and not significantly different between treatments (**Supplementary Table S1**). The pH of the potting mix ranged from 6.13 ± 0.17 to 5.24 ± 0.07 with an overall mean of 5.6 (**Supplementary Table S1**).

Plants were grown under natural light in the greenhouse at Monash University, Melbourne (37°54’28.0908’’ S and 145°8’2.2452’’ E). Three months after planting (January), the taro plants from each treatment group were transplanted to 14 L pots. Plants were grown for an additional seven months post-transplant and destructively harvested at intervals (outlined below). The mean temperature and relative humidity were 23.5/21.9°C (day/night) and 70.7/77.6% (day/night), respectively. From March, the natural photoperiod was extended to 14 hours with supplemental lighting (MK-1 Just-a14 shade, Ablite, Melbourne, Australia). Pot positions were randomized and rotated weekly to minimize microenvironment effects. Photosynthetically Active Radiation (PAR) was measured at three positions in the canopy (**Supplementary Table S2**) and the data were used to determine the PAR used in the photosynthesis measurements (see below).

### 2.2 Phenology, harvesting protocol and growth analysis

**Figure 1:**
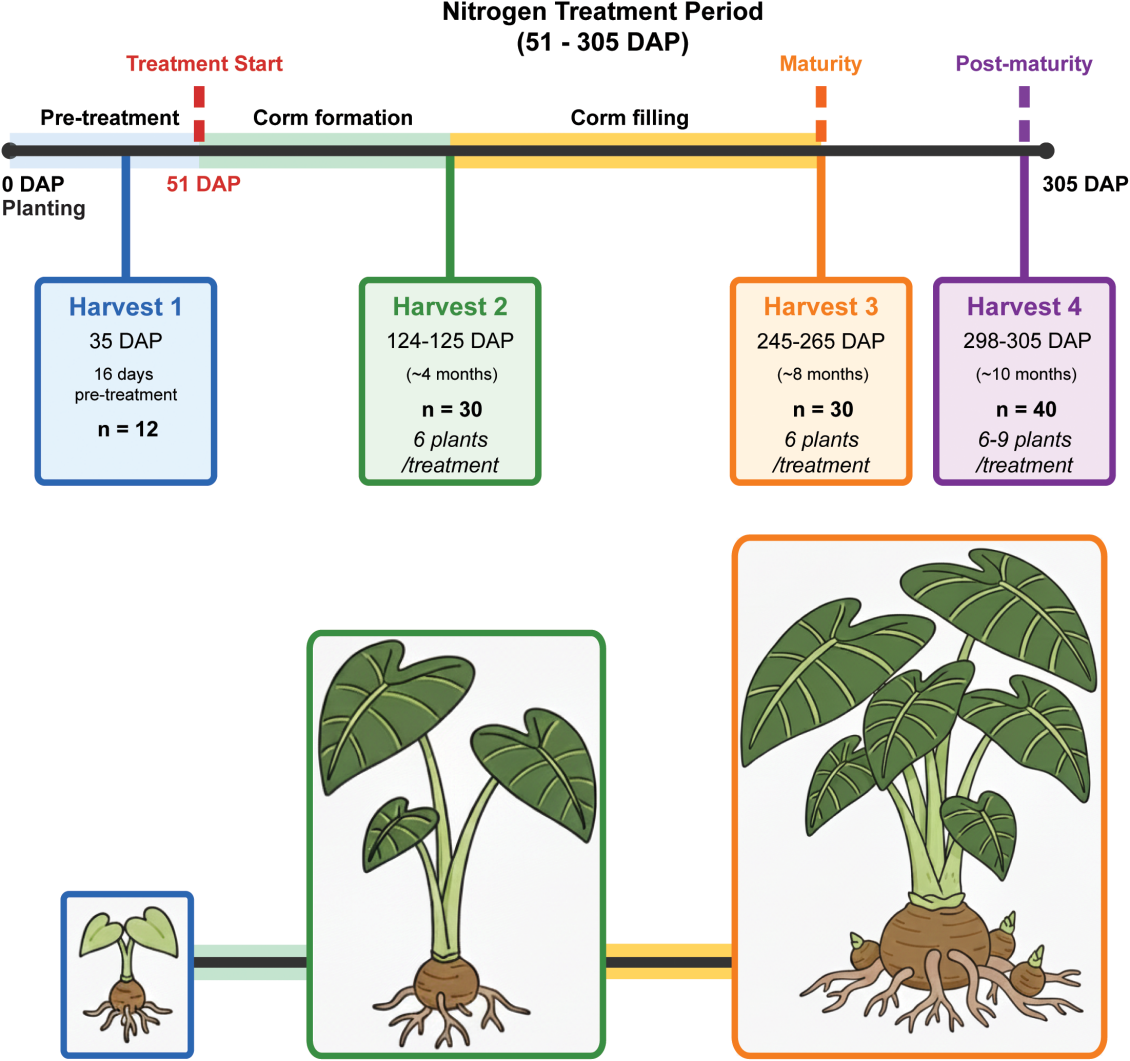
Nitrogen treatment and harvest timeline for taro plants. Harvest 1 took place 35 days after planting (DAP) and 16 days before the imposition of treatments began (n = 12). Plants were randomly assigned to one of five treatment groups, varying only in nitrogen content: 2.5, 5, 10, 15 and 20 mM N. The remaining harvests were as follows; Harvest 2, (124–125 DAP, ~4 months, n = 30, 6 plants/treatment), Harvest 3 (245–265 DAP, ~8 months, n = 30, 6 plants/treatment), and Harvest 4 (298–305 DAP, ~10 months, n = 40, 6–9 plants/treatment).

Four destructive harvests were conducted to capture different taro growth phases (**Figure 1**). Harvest 1 took place 35 days after planting (DAP) and ~2 weeks before the imposition of the treatments began (n = 12). The remaining harvests were as follows; Harvest 2 (124–125 DAP, ~4 months, n = 30, 6 plants/treatment), Harvest 3 (245–265 DAP, ~8 months, n = 30, 6 plants/treatment), and Harvest 4 (298–305 DAP, ~10 months, n = 40, 6–9 plants/treatment). The period between Harvest 1–2 spanned corm formation, Harvest 2–3 encompassed the corm filling period and Harvest 4 was post-maturity. Taro is usually harvested for consumption when the plants are at the stage captured in Harvest 3 (maturity). Plant height and leaf number were recorded prior to each harvest.

When harvesting, first the suckers (side shoots and associated cormels) were separated from the main plant. The main plant was then divided into roots, corm, petioles, and leaf blades (‘leaves’ hereafter). Leaf area of the expanded leaves was determined using a leaf area meter (LI-3000 Portable Area Meter LI-COR, Nebraska, USA). Roots, corms and cormels were washed to remove soil attached to the harvested plant material. Harvested material was placed in paper bags and oven dried at 60°C for at least one week until a stable mass was reached. Relative growth rate (RGR, g g^-1^ day^-1^) was calculated relative to the mean biomass of the main plant before treatments (Harvest 1) as follows (Equation 1):

(1) $$RGR=\frac{\left(ln\;W_{2}-ln\;W_{1}\right)}{\left(T_{2}-T_{1}\right)}$$

where: W_1_ and W_2_ are the average total plant biomass (g) at Harvest 1 (Time 1, T_1_) and the biomass of individual plants at Harvest 2, 3 or 4 (Time 2, T_2_). Time (T) is expressed in days.

In addition to the four harvests, plant height, total number of leaves and leaf area of the first fully expanded leaf were estimated every two weeks on the same plants in each treatment group. Leaf size of the first fully expanded leaf was estimated using leaf length and width at the notch following Lu et al. (2004) as modified by Crimp et al. (2017). Additional phenological data are available on-line at the Monash Bridges (DOI: 10.26180/30423973).

### 2.3 Photosynthetic parameters

The rate of photosynthesis (A) was measured on the youngest fully expanded leaf of each plant (n = 5-7) in the week prior to the Harvest 3 (20–27 June 2015). Measurements were taken with a LI-COR LI-6400 Portable Photosynthesis Machine (LI-COR Nebraska, USA) under glasshouse conditions as follows; relative humidity = 55-60%, temperature = 25°C, CO_2_ = 400 ppm, and PAR = 500 µmol quanta m^-2^ s^-2^ (with 10% blue light). Measurements were taken from the middle of the side of the lamina, avoiding the midrib and prominent veins. Additional environmental data are available on-line at the Monash Bridges (DOI: 10.26180/30423973).

### 2.4 Nutrient analysis and nitrogen use efficiency

The oven-dried (60°C) samples of a subset of leaves and corms from Harvest 3 (245–265 DAP) were ground to a fine powder for nutrient analysis. Total elemental N and Carbon (C) were determined using a LECO CNS2000 analyzer (Monash University) (n = 4). Other elements (P, K, S, Ca, Mg, Na, Cu, Zn, Mn, Fe, Bo, Mo, Co, Si) were measured on microwave-digested samples using inductively coupled plasma mass spectrometry (ICPMS; Environmental Analysis Laboratories, Southern Cross University, NSW) (n = 3). Protein was estimated using total N and S concentrations.

Nitrogen use efficiency (NUE) is reported using the following two NUE indicators (Equations 2, 3, adapted from (Antille and Moody, 2021).

(2) $$AFNR=\frac{\left(Corm\;biomass\times [N]\right)}{N_{R}}\;\;$$

where AFNR is the apparent fertilizer nitrogen (N) recovery in corm (kg kg^-1^) and the corm biomass is the corm dry matter (expressed as kg per pot). [N] is the nitrogen concentration in the corm (%, w/w) and N_R_ is the nitrogen application rate (expressed as kg per pot).

(3) $$PFP_{N}=\frac{Corm\;biomass}{N_{R}}$$

where corm biomass and N_R_ are as defined in Equation 2.

While both NUE expressions are similar, Equation 3 denotes the amount of nitrogen taken up by the corm from a specified rate of applied N fertilizer, whereas Equation 2 denotes dry matter accumulated by the corm at harvest (yield) from a specified rate of applied N fertilizer.

### 2.5 Statistical analysis

Statistical analyses were undertaken with GenStat Release 22^nd^ Edition (VSN International Ltd., 2025). ANOVA was used to compare treatments within harvests. The least significant differences (LSD) were used to compare means with a probability level of 5% (p < 0.05). Statistical analyses were graphically assessed by means of residual plots and normalization of the data was not required, unless otherwise stated. Tukey’s HSD tests were performed using RStudio (Version 0.99.446) and used to detect significant differences between treatment means (p < 0.05). Values in the text, tables and graphs are Means ± 1 Standard Error, unless otherwise stated.

## 3 Results

### 3.1 Phenology, leaf area and photosynthesis

**Figure 2:**
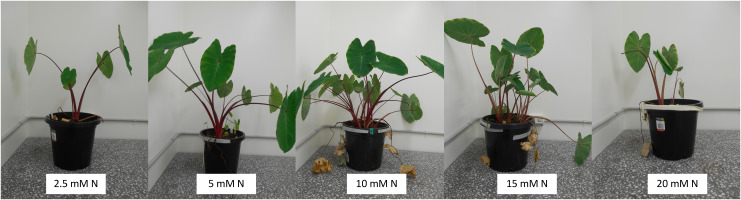
Taro plants (*Colocasia esculenta*) supplied with fertilizer containing five different concentrations of nitrogen (2.5, 5, 10, 15, 20 mM N) at Harvest 3 (245–265 DAP), approx. 7 months after treatments began.

Leaf number and plant height were relatively consistent between treatment groups over time. (**Figure 2**; **Table 1**; **Supplementary Table S3**). Across harvests, leaf area of fully expanded leaves was highest in 15mM N-treated plants at Harvest 2 (H2, during corm filling) with an area of 1055.9 ± 133.5 cm^2^, compared to 259.98 ± 69.52 cm^2^ in plants from the same treatment at Harvest 4 (H4, Post maturity) (**Table 1**). In contrast, the total leaf area of plants from the lowest, 2.5 mM N treatment group was significantly smaller than that of the 15 mM N-treated plants at H2 (509.81 ± 59.79 cm^2^) but larger at H4 (689.00 ± 171.20 cm^2^).

Table 1Total biomass (dry weight, g dw), Relative Growth Rate (RGR), height, leaf area, leaf number, and number of suckers for taro plants from five different nitrogen treatment groups (2.5, 5, 10, 15 and 20mM N) harvested at intervals (Harvests 1-4).

**(a):** Harvest 1: approx. 2 weeks before treatments began (35 DAP).

Total biomass (g dw)	4.91 ± 0.52
Height (cm)	31.2 ± 1.32
Leaf number	3.25 ± 0.20
Number of suckers	0

**(b):** Harvest 2: approx. 2.5 months after treatments began (124–125 DAP).

	2.5 mM	5 mM	10 mM	15 mM	20 mM	p value
Total biomass (g dw)	20.70 ± 2.93	24.52 ± 8.69	27.11 ± 5.93	28.85 ± 2.86	17.20 ± 2.44	0.510
RGR (g g^-1^ day^-1^)	0.0068 ± 0.0006	0.0063 ± 0.0018	0.0079 ± 0.0009	0.0085 ± 0.0006	0.0058 ± 0.0009	0.359
Height (cm)	49.1 ± 3.1	52.9 ± 3.8	53.0 ± 2.5	56.8 ± 2.2	55.3 ± 2.2	0.390
Leaf area (cm^2^)	509.81 ± 59.79^A^	656.03 ± 143.33^AB^	768.02 ± 106.25^BC^	1055.55 ± 133.51^C^	476.50 ± 103.39^A^	0.010
Leaf number	3.2 ± 0.2^A^	3.2 ± 0.3^A^	3.8 ± 0.3^AB^	4.2 ± 0.3^B^	3.2 ± 0.2^A^	0.027
Number of suckers	0.0	0.5 ± 0.5	0.3 ± 0.3	0.5 ± 0.3	0.2 ± 0.2	0.759

**(c):** Harvest 3: approx. 7 months after treatments began (245–265 DAP).

	2.5 mM	5 mM	10 mM	15 mM	20 mM	p value
Total biomass (g dw)	49.72 ± 7.72^A^	113.17 ± 5.18^B^	115.89 ± 24.38^B^	60.91 ± 4.20^A^	39.32 ± 10.64^A^	<0.001
RGR (g g^-1^ day^-1^)	0.0043 ± 0.0003^A^	0.0059 ± 0.0001^B^	0.0058 ± 0.0004^B^	0.0048 ± 0.0001^AB^	0.0036 ± 0.0005^A^	<0.0001
Height (cm)	52.5 ± 1.9	51.9 ± 4.8	58.3 ± 4.8	47.5 ± 3.0	44.3 ± 5.3	0.202
Leaf area (cm^2^)	696.95 ± 156.58^A^	381.95 ± 66.45^BC^	496.28 ± 125.35^BC^	196.06 ± 41.89^D^	270.08 ± 71.79^CD^	0.015
Leaf number	2.3 ± 0.3	2.2 ± 0.31	4.0 ± 1.5	2.3 ± 0.6	1.7 ± 0.3	0.271
Number of suckers	2.7 ± 0.9^A^	5.00 7± 0.7^AB^	7.3 ± 0.8^B^	5.50 ± 0.6^B^	3.5 ± 1.2^A^	<0.001

**Table:** **(d)** Harvest 4: approx. 8.5 months after treatments began (298–305 DAP).

	2.5 mM	5 mM	10 mM	15 mM	20 mM	p value
Total biomass (g dw)	74.77 ± 10.12^A^	147.32 ± 10.93^BC^	168.72 ± 13.78^C^	102.91 ± 11.03^AB^	66.49 ± 12.58^A^	<0.0001
RGR (g g^-1^ day^-1^)	0.0043 ± 0.0002^A^	0.0055 ± 0.0001^B^	0.0057 ± 0.0001^B^	0.0048 ± 0.0002^A^	0.0041 ± 0.0003^A^	0. 004
Height (cm)	58.4 ± 1.9	61.6 ± 2.6	51.1 ± 6.3	56.8 ± 3.9	44.1 ± 3.9	0. 067
Leaf area (cm^2^)	689.00 ± 171.20^A^	498.90 ± 100.10^A^	170.94 ± 44.40^B^	259.98 ± 69.52^AB^	41.88 ± 20.55^C^	0.001
Leaf number	2.3 ± 0.7	1.7 ± 0.4	2.3 ± 0.6	2.2 ± 0.5	2.5 ± 0.6	0.926
Number of suckers	2.7 ± 0.6^A^	6.1 ± 0.4^BC^	8.0 ± 0.5^C^	5.9 ± 0.8^B^	5.0 ± 0.7^AB^	0.016

RGR is calculated relative to Harvest 1 (35 DAP, two weeks before treatments were imposed). Leaf area is the area of fully expanded leaves only. Means are the average of 6–9 replicates ± 1 SE. Means in rows with the same letter superscript are not significantly different at p < 0.05. See **Figure 2** and **Supplementary Table S3** for additional data.

Assimilation rates were measured in the week prior to Harvest 3 when plants were ~7 months old. Photosynthetic rates ranged from 4.90 ± 0.62 µmol m^-2^ s^-1^ to 6.55 ± 0.68 µmol m^-2^ s^-1^ in plants from the 2.5 mM and 15 mM N treatments; however, no significant differences were detected (**Table 2**).

**Table 2:** Assimilation rate as average μmol CO_2_ m^-2^s^-1^ for the first fully expanded leaf per plant for each of the five nitrogen treatment groups at ~250 DAP.

N treatment	µmol CO_2_ m^-2^s^-1^
2.5 mM	6.55 ± 0.68
5 mM	6.12 ± 0.68
10 mM	5.23 ± 0.80
15 mM	4.90 ± 0.62
20 mM	4.97 ± 0.29

Each mean is the average of 5–7 replicates ± 1 SE. Means are not statistically significant (p > 0.05).

### 3.2 Growth and biomass partitioning

**Figure 3:**
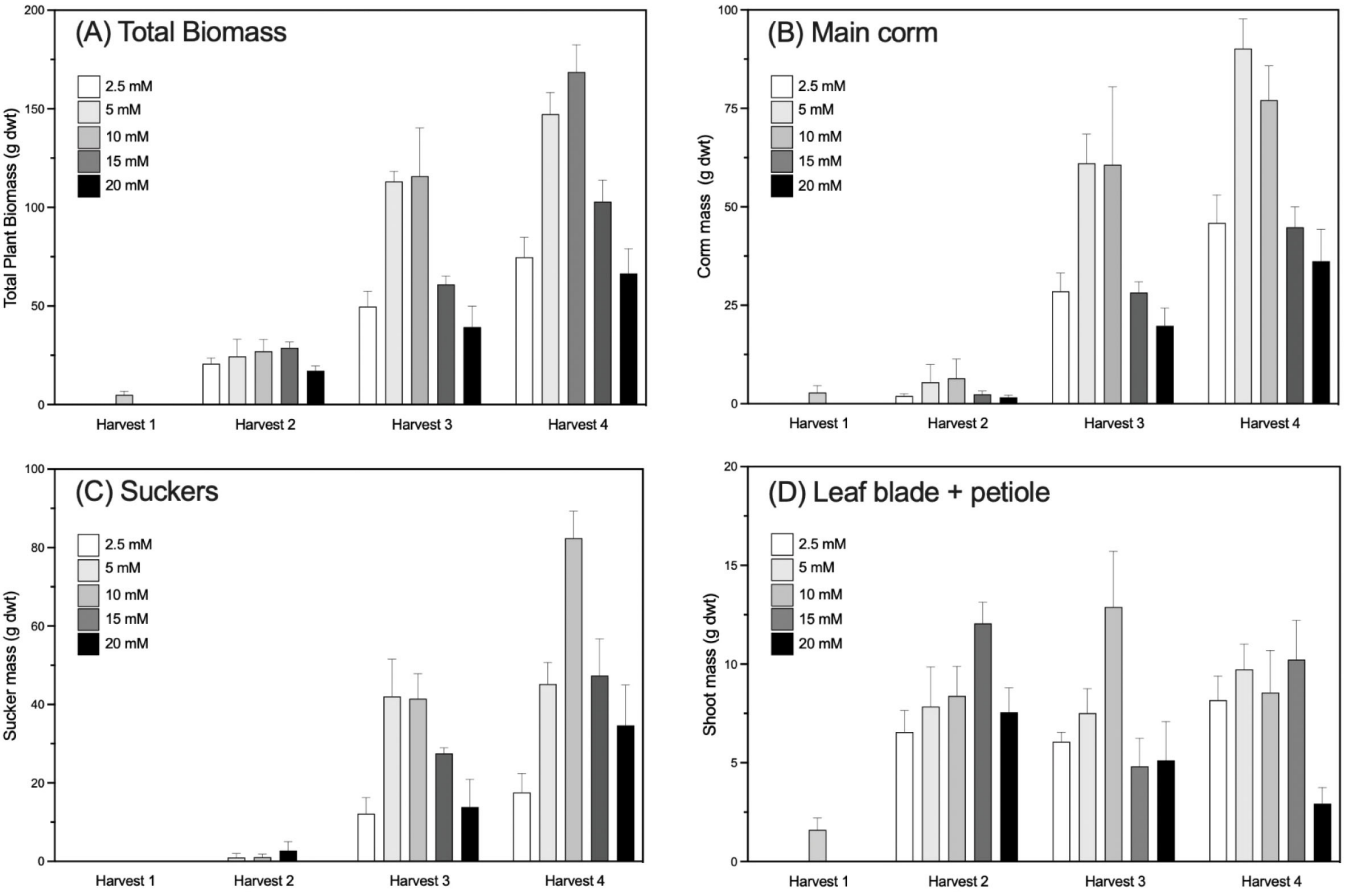
Mean dry biomass of taro plants (*Colocasia esculenta*) grown at five different concentrations of nitrogen and destructively harvested at intervals. **(A)** Total Biomass, **(B)** Mass of the corm on the main plant, **(C)** Mass of all suckers (side shoots) including cormels, **(D)** Shoot mass (leaf blade plus petiole biomass). Four harvests were conducted: Harvest 1 (35 DAP and before treatments began); Harvest 2 (124–125 DAP); Harvest 3 (245–265 DAP); and Harvest 4 (298–305 DAP). Values are the mean of at least 6 replicates ± 1 SE. Additional data on plant growth can be found in **Table 1** and **Supplementary Table S3**.

Total biomass increased for each treatment group over the duration of the study, but the rate of increase and the partitioning between different organs varied significantly with the amount of N supplied (**Figure 3**; **Table 1**; **Supplementary Table S3**). By the third harvest (H3, ~7 months after treatments began), the RGR was significantly higher in plants from the 5 mM and 10 mM N treatment groups (p < 0.001) and lowest in plants grown at 2.5 mM and 20 mM N (**Figure 2**).

At the first harvest, 1 month after planting and before treatments commenced, more than half of the plant biomass was in the developing corm (**Figure 3**; **Table 1A**). Over the next two months the proportion of biomass in the corm of the main plant decreased, with plants in all treatment allocating greater biomass to the leaves and petioles (**Figure 3**; **Table 1B**). As plants neared maturity, resource allocation reverted to corm biomass, with ~50% of biomass attributed to the corm by Harvests 3 and 4. Corm biomass of the main plant was highest in absolute terms and as a proportion of total biomass in 5 mM and 10 mM N-treated plants at all post-treatment harvests (p < 0.05). Plants grown at 2.5 mM N were smaller and had the highest proportion of biomass allocated to the roots.

There were clear differences in the number and mass of the suckers, with significant differences across treatment and time of harvest (**Figure 3C**; **Table 1**). There were no suckers or cormels on young plants at the first harvest (35 DAP), reaching 2–10 cormels per plant at Harvest 3 (~250 DAP), depending on N level. Cormel biomass similarly varied with treatment and time. At Harvest 3, when plants were at the typical age for harvesting, sucker biomass was significantly higher in the 5 mM and 10 mM N groups (42.06 ± 9.48 and 41.47 ± 6.42 g) compared to the 2.5 mM N group (12.18 ± 4.05 g).

### 3.3 Nutrient analysis

**Figure 4:**
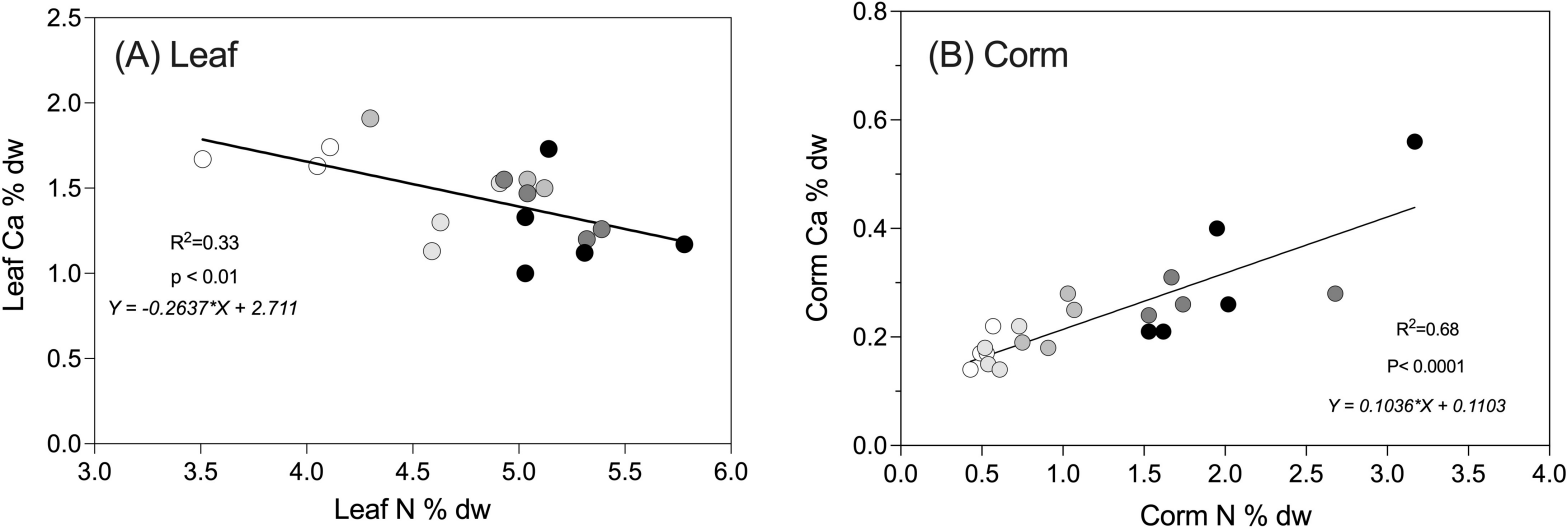
Calcium concentration plotted relative to total nitrogen for **(A)** Leaf and **(B)** Corm of taro plants (*Colocasia esculenta*) grown at five different concentrations of nitrogen (2.5, 5, 10, 15, 20 mM N). Values represent one plant and are expressed as % dry weight (dw). Data are for Harvest 3 (245–265 DAP). Regression lines are significant (p < 0.01).

Elemental analysis was performed on the leaves and corms of the main plant from Harvest 3, when plants were at maturity (~250 DAP) (**Table 3**). Nitrogen was the only element where highly significant differences between treatments were detected in both leaves and corms: foliar elemental nitrogen increased with increasing N supply from 3.89 ± 0.19% to 5.26 ± 0.14% (p < 0.001) (**Figure 4**; **Table 3A**). The increase in tissue nitrogen concentration with treatment was even more pronounced in the corms, increasing four-fold from 0.51 ± 0.03% to 2.06 ± 0.29% with increasing N supply (**Table 3B**; p < 0.001). Elemental ratios of physiological interest (N:P and C:N) reflected the differences in tissue nitrogen. The C:N ratio was significantly higher in leaves from the 2.5 mM N treatment group (p < 0.001), while in corms C:N ratios were higher in both the 2.5 and 5 mM N treatment groups (p < 0.0001). The N:P ratios in the leaves were not significantly different between treatments, but corm N:P was nearly four times greater in plants grown 20 mM N compared to those grown at 2.5 mM N (p < 0.001).

**Table 3A:** Concentration of macro- (% dry weight, dw) and micronutrients (mg kg^-1^ dw) in the expanded leaves of plants from five different nitrogen treatment groups (2.5, 5, 10, 15 and 20mM N).

Macronutrients	N treatment					p value
	2.5 mM	5 mM	10 mM	15 mM	20 mM	
Macronutrients (% dw)						
Nitrogen	3.89 ± 0.19^A^	4.71 ± 0.10^AB^	4.75 ± 0.20^B^	5.17 ± 0.11^B^	5.26 ± 0.14^B^	<0.001
Phosphorus	0.35 ± 0.04	0.37 ± 0.03	0.30 ± 0.03	0.39 ± 0.02	0.38 ± 0.04	0.301
Potassium	3.64 ± 0.39	4.26 ± 0.21	3.63± 0.38	4.06 ± 0.17	4.05 ± 0.19	0.472
Sulphur	0.31 ± 0.01^A^	0.35 ± 0.01^AB^	0.40 ± 0.03^B^	0.41 ± 0.01^B^	0.39 ± 0.01^AB^	0.025
Carbon	41.10 ± 0.44	41.61 ± 0.46	41.11 ± 0.24	41.75 ± 0.48	41.94± 0.28	0.395
Calcium	1.68 ± 0.03	1.32 ± 0.12	1.65 ± 0.13	1.37 ± 0.08	1.27 ± 0.13	0.068
Magnesium	0.32 ± 0.02	0.34 ± 0.01	0.42 ± 0.03	0.39 ± 0.02	0.34 ± 0.02	0.060
Sodium	0.00	0.02	0.03 ± 0.01	0.02	<0.01	N/A
Micronutrients (mg kg^-1^)						
Copper	5.94 ± 0.73	5.26 ± 0.30	5.66 ± 2.08	5.89 ± 0.37	5.35 ± 0.48	0.999
Zinc	20.83± 3.17	31.05 ± 1.47	24.39 ± 3.98	29.16 ± 1.32	34.73 ± 7.67	0.492
Manganese	165.17 ± 4.85	88.91 ± 18.87	95.51 ± 14.87	73.78 ± 9.60	63.25 ± 6.69	0.892
Iron	104.66 ± 12.19	101.48 ± 1.90	114.49 ± 17.20	115.76 ± 3.33	128.68 ± 12.51	0.538
Boron	50.24 ± 9.84	65.95 ± 9.26	65.11 ± 12.33	57.71 ± 2.48	43.37 ± 4.05	0.224
Molybdenum	2.60 ± 0.88	1.51 ± 0.14	2.24 ± 0.19	1.54 ± 0.14	1.970 ± 0.15	0.225
Cobalt	0.00	<0.01	0.33	<0.01	<0.01	N/A
Silicon	259.22 ± 9.02	270.27 ± 3.67	242.92 ± 23.30	295.41± 32.97	323.42 ± 32.45	0.263
Elemental ratios						
C:N	10.62 ± 0.66^A^	8.85 ± 0.24^B^	8.70 ± 0.34^B^	8.08 ± 0.18^B^	8.0 ± 0.24^B^	<0.001
N:P	11.23 ± 0.83	12.98 ± 1.04	16.78 ± 2.09	13.28 ± 0.76	14.06 ± 1.05	0.117
Crude protein (%)	24.33 ± 1.19^A^	29.43 ± 0.63^B^	29.69 ± 1.23^B^	32.31 ± 0.69^B^	32.85 ± 0.87^B^	<0.001

Each mean is the average of four replicates ± 1 SE. Means in rows with the same letter superscript are not significantly different (p > 0.05). N/A indicates where it was not possible to calculate probabilities as values were below the limit of detection.

**Table 3B.** Concentration of macro- (% dry weight) and micronutrients (mg kg^-1^ dw) in the main corms of plants from five different nitrogen treatment groups (2.5, 5, 10, 15 and 20mM N).

Macronutrients	N Treatment					
	2.5 mM	5 mM	10 mM	15 mM	20 mM	p value
Macronutrients (% dw)						
Nitrogen	0.51 ± 0.03^A^	0.60 ± 0.05^A^	1.18 ± 0.09^AB^	1.91 ± 0.26^B^	2.06 ± 0.29^B^	<0.001
Phosphorus	0.16 ± 0.02	0.15 ± 0.01	0.09 ± 0.01	0.19 ± 0.04	0.17 ± 0.04	0.118
Potassium	1.25 ± 0.15	1.31 ± 0.13	1.30 ± 0.14	1.48 ± 0.13	1.15 ± 0.05	0.471
Sulphur	0.09 ± 0.00^A^	0.09 ± 0.00^A^	0.14 ± 0.02^AB^	0.17 ± 0.03^AB^	0.19 ± 0.02^B^	0.012
Carbon	40.67 ± 0.31	40.12 ± 0.08	39.54 ± 2.02	41.66 ± 0.24	41.76 ± 0.39	0.506
Calcium	0.18 ± 0.02^A^	0.17 ± 0.02^AB^	0.22 ± 0.02^AB^	0.27 ± 0.01^AB^	0.33 ± 0.07^B^	0.050
Magnesium	0.12 ± 0.01	0.12 ± 0.01	0.21 ± 0.06	0.16 ± 0.02	0.17 ± 0.02	0.414
Sodium	0.02 ± 0.00^A^	0.04 ± 0.01^A^	0.15 ± 0.08^AB^	0.17 ± 0.01^AB^	0.24 ± 0.04^B^	0.016
Micronutrients (mg kg^-1^)						
Copper	3.86 ± 0.40	2.72 ± 0.34	7.31 ± 4.59	4.03 ± 1.13	5.46 ± 1.75	0.740
Zinc	26.74 ± 1.47	21.75 ± 4.43	18.33 ± 7.37	11.94 ± 3.15	15.04 ± 4.08	0.306
Manganese	21.27 ± 2.52	17.92 ± 4.33	16.07 ± 2.88	19.28 ± 1.56	19.42 ± 4.12	0.892
Iron	53.76 ± 2.09	55.92 ± 2.98	64.97 ± 4.51	63.82 ± 5.61	79.62 ± 18.24	0.297
Boron	5.22	5.65 ± 0.14	14.99 ± 8.68	7.27 ± 0.86	8.61 ± 1.64	N/A
Molybdenum	0.61 ± 0.08	0.43 ± 0.10	0.57 ± 0.19	0.97 ± 0.28	1.21 ± 0.44	0.327
Cobalt	<0.01	<0.01	0.28	<0.01	0.14 ± 0.02	N/A
Silicon	143.57 ± 12.05	152.31 ± 6.03	164.06 ± 6.48	168.40 ± 3.65	135.18 ± 18.35	0.145
Elemental ratios						
C:N	81.21 ± 4.27^A^	68.38 ± 5.12^A^	37.08 ± 4.32^B^	22.88 ± 2.45^BC^	21.60 ± 2.32^C^	<0.0001
N:P	3.09 ± 0.18^A^	3.96 ± 0.26^A^	12.80 ± 8.54^B^	10.57 ± 0.56 ^AB^	12.90 ± 1.38^B^	<0.001
Crude protein (%)	3.16 ± 0.18^A^	3.73 ± 0.29^AB^	7.37 ± 1.54^BC^	11.91 ± 1.64^CD^	12.86 ± 1.84^D^	<0.001

Each mean is the average of four replicates ± 1 SE. Means in rows with the same letter superscript are not significantly different (p > 0.05).

Corm calcium (Ca) concentration was significantly higher in 20mM N-treated plants compared to 2.5mM N-treated plants (p = 0.050). A highly significant correlation was present between leaf nitrogen and leaf calcium (**Figure 4A**; p < 0.001). In contrast, corm N and Ca concentrations were negatively correlated (**Figure 4B**). Additionally, Sulphur (S) concentration was highest in the leaves of plants grown in the middle treatments (**Table 3A**; 5–15 mM N; p = 0.025) and sodium (Na) concentration increased in corms with increasing levels of N in the nutrient solution (**Table 3B**; p = 0.002). No treatment effect was detected in potassium (K) concentrations of leaves or corms.

### 3.4 Nitrogen use efficiency indices

**Figure 5:**
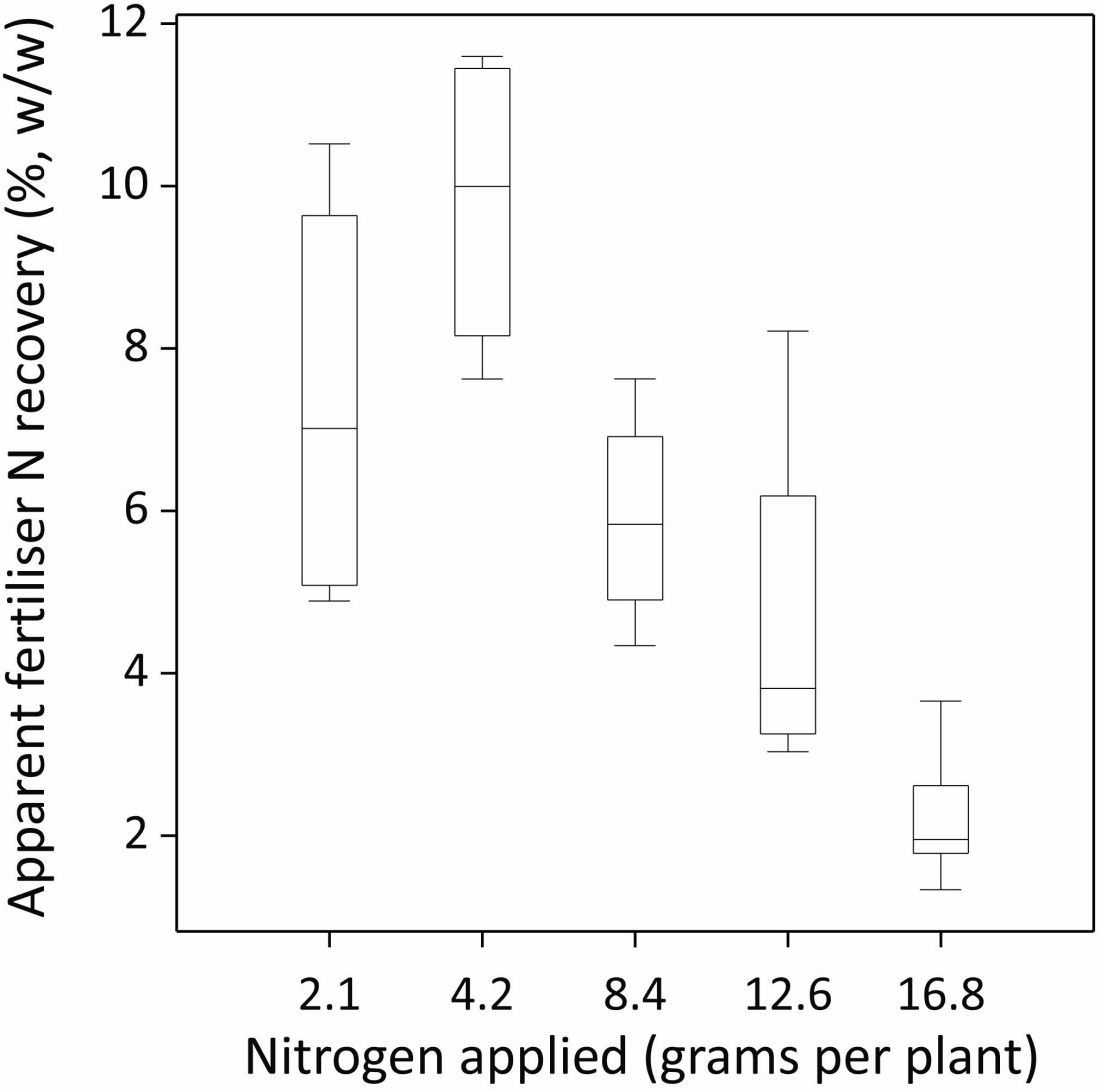
Nitrogen use efficiency (NUE) expressed as the apparent fertilizer nitrogen (N) recovery (AFNR) in corm biomass (%, w/w) as a function of N applied (g N per plant or pot). The box spans the interquartile range of the values in the variate (Q3–Q1). Whiskers extend to the most extreme data values within the inner ‘fences’, which are at 1.5 times the interquartile range beyond the quartiles (or the maximum value where that is smaller).

**Figure 6:**
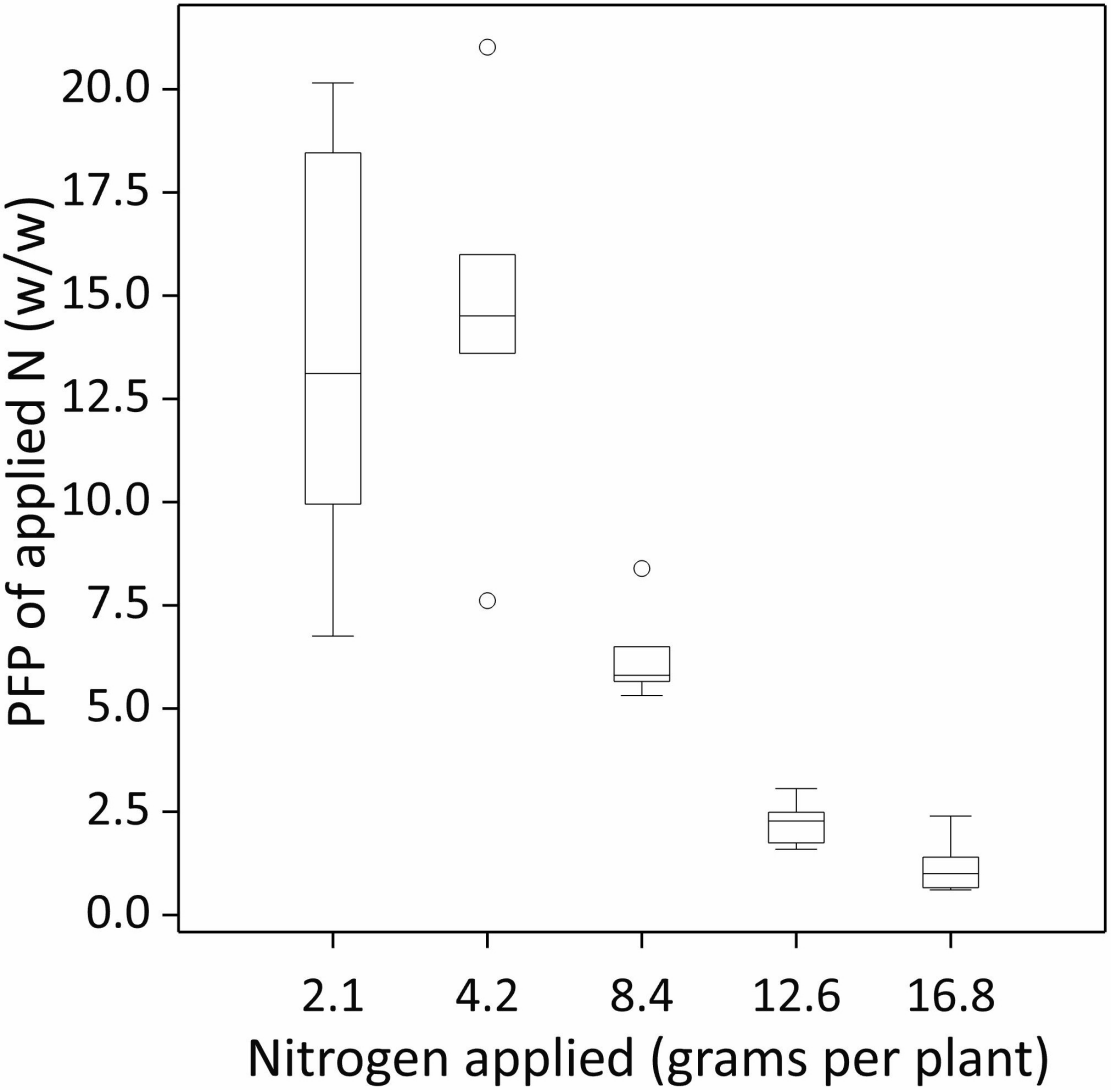
Nitrogen use efficiency expressed as the partial factor productivity of applied fertilizer N (PFP_N_, w/w) as a function of N applied (g N per plant or pot). The box spans the interquartile range of the values in the variate (Q3–Q1), with the middle line indicating the median (Q2). Whiskers extend to the most extreme data values within the inner ‘fences’, which are at 1.5 times the interquartile range beyond the quartiles (or the maximum value if that is smaller). Individual outliers are identified with a white circle.

There were significant N application rate effects on both NUE indicators; the applied fertilizer N recovery (AFNR) in corm biomass and the partial factor productivity of applied N (PFP_N_) (**Figures 5**, **6**; **Table 4**; p-values < 0.001). On average, AFNR and PFP_N_ were the highest when N was applied at a rate of 4.2 g per pot (9.80%, w/w and 14.54 kg kg^-1^, respectively). NUE decreased when N was applied either above or below 4.2 g per pot. Across all N application rates, the AFNR and PFP_N_ were 6% (w/w) and 7.56 kg kg^-1^, respectively (**Table 4**).

**Table 4:** The Apparent Fertilizer Nitrogen Rate (AFNR) and Partial Factor Productivity of applied N (PFP_N_) for taro main plants calculated using the biomass at Harvest 3 (245–265 DAP), the total amount of nitrogen applied between Harvests 1 and Harvest 3, and (for AFNR) corm nitrogen concentration.

N treatment	AFNR %	PFP_N_ g g^-1^ se	N applied (g)
2.5 mM	7.36 ± 1.11	13.59 ± 2.21	2.1
5 mM	9.80 ± 0.80	14.54 ± 1.76	4.2
10 mM	5.91 ± 0.62	6.23 ± 0.55	8.4
15 mM	4.72 ± 0.97	2.24 ± 0.22	12.6
20 mM	2.23 ± 0.35	1.18 ± 0.27	16.8

Values are means of all replicate plants ± 1 SE.

## 4 Discussion

The key to future food security may lie in the untapped potential of widely grown but under-researched crops, such as taro (Matthews and Ghanem, 2021; Aditika et al., 2022; Lebot and Ivančič, 2022). Fundamental to this is the development of crop recommendations to recover fertilizer in harvested plant material and maximize the economic return from applied nutrients. In doing so, the risk of nutrient losses to the environment due to poor use efficiency is minimized (Antille et al., 2023). Here, the response of taro to different applications of N fertilizer (ranging from 2.5mM to 20 mM N) was assessed under controlled environmental conditions to determine optimum levels for plant growth, biomass partitioning and indicators of nutritional composition. The work aids the development of management strategies aimed at optimizing yields, improving nitrogen use efficiency and generating baseline data for further nutrient experiments.

### 4.1 N supply effects on growth are driven by leaf area rather than photosynthesis

Nitrogen is the most limiting nutrient in most non-fertilized systems (Zierer et al., 2020). We hypothesized that growth and photosynthetic rate would be highest in plants receiving nutrient solutions with the highest concentration of nitrogen (Hypothesis 1). This hypothesis was not supported, as total biomass and relative growth rates were optimal when plants were supplied with mid-levels of N (5–15 mM N). Further increases in N supply did not translate into additional biomass (**Figure 3A**).

Growth rates appeared to be primarily driven by differences in the total area of expanded leaves rather than assimilation rates (**Table 1**). Rates of photosynthesis on a per area basis in leaves of the same age tended to increase with N concentration but the differences were not significant (**Tables 1**, **2**). Leaf area was estimated using the leaf width and length of fully expanded leaves and, although this was not an absolute measure, the differences between groups were consistent with published work on taro (Jacobs and Clarke, 1993; Vos and van der Putten, 1998; Mergedus et al., 2015). While it is possible that photosynthetic rates may have differed in plants at other stages of development, the large treatment effects on total leaf area are likely to be the dominant influence. Profuse leaf area is common in taro plants grown at high nitrogen, whereas lower levels of nitrogen are optimal for maximal corm growth (Hartemink et al., 2000). Here, the highest area of expanded leaves in plants was from the 15 mM N treatment at Harvest 2, during the early stages of corm filling, supporting the observations of Jacobs and Clarke (1993) that higher nitrogen availability benefits leaf size in the early stage of growth. At the final harvest (H4), expanded leaf area was highest in 2.5 mM N-treated plants, greater than that of 5–15 mM N-treated plants, which were entering or past maturity (**Table 1**; **Supplementary Table S3**). The limited availability of nitrogen in the 2.5mM N treatment group likely resulted in developmental delays that favored allocation of resources to vegetative growth (Craswell et al., 1996).

### 4.2 Early biomass partitioning patterns are treatment-dependent and linked to N ratios

Differences in biomass partitioning between roots, shoots, corms and the growth of suckers were established early in development (**Table 1**; **Supplementary Table S3**). Plants from the 2.5 mM and 5mM N treatment groups showed the greatest allocation of resources to the roots. This is not surprising since plants that are nitrogen deficient often priorities root growth in an attempt to improve nutrient uptake (Hermans et al., 2006). Taro root systems develop substantially in the early stage of plant growth in order to facilitate the uptake of essential nutrients (Goenaga, 1995; Lebot, 2009). There were no suckers or cormels on young plants at the first harvest (35 DAP), while plants at Harvest 3 (~250 DAP) had 2–10 cormels. Plants grown at 2.5 mM N had the fewest suckers, consistent with Hypothesis 3, likely due to low assimilate supply and nitrogen deficiency (**Tables 1**, **3**).

As hypothesized (Hypothesis 2), corm biomass (absolute and proportional) was highest at mid-level N (5–15 mM), with some maturity-dependent variation (**Figure 3**; **Supplementary Table S3**). In the field, corm formation peaks at 6-8 months after planting and then decreases (Onwueme, 1999; Lebot, 2009), with harvests coinciding with maximum corm mass. Corms of plants grown at 2.5 mM N developed slowly, with very little increase in the early corm development phase. The lower concentrations of tissue N and high C:N ratios in these plants (**Table 3**) are indicative of a N deficiency, leading to delayed development (Reuter and Robinson, 1997; Zierer et al., 2020). Corm growth was also inhibited in plants grown at the highest level of N (20 mM N). In potato, tuber development is suppressed at high N supply (Hartemink et al., 2000; Zierer et al., 2020). The mechanism for this is thought to be linked to the inhibition of gibberellic acid (GA) production when tissue N is high relative to C, which prevents tuber induction, initiation and growth (Abelenda et al., 2011). Plants grown at 20 mM N had very low C:N ratios in leaves and corms (i.e., relatively high N, **Table 3**), which may account for the low corm biomass, however, shoot growth was also stunted. In Samoa, an imbalance between N and P has also been reported to affect taro growth (Antille et al., 2023). Here, higher N:P ratios were detected in the corms 20 mM N-treated plants, suggesting that N was vastly in excess of P during the corm filling period and may have adversely affected growth. However, the small, non-significant difference in N relative to P with increasing N supply in the leaves (**Table 3A**) was unlikely to have affected assimilation or growth overall (**Table 3A**).

Another explanation for the reduced growth of corms in plants grown at 20 mM N could be the sensitivity of taro to ammonia (NH_4_^+^). However, the fixed proportion of the nitrogen supplied as NH_4_^+^ in our nutrient solution here was only 16%, and Osorio et al. (2003) found no detrimental effect on growth or yield with NH_4_^+^ as high as 50%. High NH_4_^+^ concentrations also reduce pH, which has the potential to affect the uptake of nutrients necessary for root growth and development, such as P (pH 5.5-6.5) and N (pH 6.0-8.0) (Deenik et al., 2013). The optimum soil pH for taro is between 6.0 and 7.0 (Deenik et al., 2013). Lower soil pH may restrict the healthy growth and development of taro root systems and consequently limit biomass production. In this study, soil pH was approximately one point lower, ranging from 6.1 in soil sampled from pots in the lowest 2.5 mM N treatment group down to 5.2 in soil sampled from pots in the 20 mM N treatment group (**Supplementary Table S1**). As such, lower soil pH may have limited root growth and development in 20 mM N-treated plants.

### 4.3 Nutrient composition varied with plant size, nutrient status and minor allocation trade-offs

Macro- and micronutrient concentrations were largely typical of the expected range for taro and storage organ crops, with higher concentrations in the leaves than in the corms (Craswell et al., 1996; Reuter and Robinson, 1997; Osorio et al., 2003; Mergedus et al., 2015; Lloyd et al., 2021; Aditika et al., 2022). All indicators were consistent with those of well-fertilized plants with no signs of deficiencies (Reuter and Robinson, 1997), except for signs of nitrogen limitation in the 2.5 mM N-treated plants (**Table 3A**).

Taro contains raphides of calcium oxalate (CaOx), which must be removed by washing before consumption (Kristl et al., 2021). Hypothesis 4 proposed that calcium oxalate per mass would be higher when growth was limited by N, consistent with plant defense-growth theories (Herms and Mattson, 1992). Lloyd et al. (2021) found higher concentrations of both Ca and Oxalate in plants whose growth was stunted at high salinity. Here, Ca decreased from 1.68% to 1.27% with increasing N in leaves (**Table 3A**; p = 0.068), consistent with our hypothesis and similar to the results reported by Sunell and Healey (1985). However, unlike Sunell and Healey (1985) and contrary to our hypothesis, corm Ca increased significantly (from 0.18 to 0.33%) with increasing N (**Table 3B**; p = 0.05). It could be that rather than allocation of resources to CaOx (i.e. defense) reducing with increased resource supply, as proposed by Herms and Mattson (1992), plants instead diverted excess resources to Ca and oxalate during the latter stages of corm filling. Bradbury and Holloway (1988) and Lebot (2009) report much higher concentrations of calcium in the corms than observed here, ranging from 0.5 to 4%. Both measurements were made on field grown taro and it is possible that more variable conditions could drive the allocation of resources to defense. In several other species unrelated to taro, CaOx is stored and remobilized as an internal source of carbon under various environmental conditions (Tooulakou et al., 2016). It is possible that taro similarly stores CaOx in reserve when the requirements for growth are met at higher levels of N supply. It is worth noting that high NH_4_^+^ can result in reduced uptake or translocation of cations, especially Ca^2+^, Mg^2+^, Mn^2+^ (Osorio et al., 2003). Given the relatively high levels of NH_4_^+^ in the 15 and 20 mM N nutrient solutions used here, any direct effect on CaOx may have been masked. While not definitive, the results presented here indicate that breeding for low CaOx is likely preferrable to attempting to manipulate raphide production via nutrient supply (Lebot and Ivančič, 2022).

Many edible aroids are known to be cyanogenic (Gleadow and Møller, 2014). Bradbury et al. (1995) recorded low levels of the cyanogenic glucoside triglochinin in taro and several other yams. However, no hydrogen cyanide was detected in the leaves or corms of the taro plants in this study, consistent with Lloyd et al. (2021).

### 4.4 Nitrogen use efficiency and implications for agricultural systems

Nitrogen use efficiency calculations showed relatively low N recoveries in corm biomass (< 10% w/w) and low conversion efficiency of N applied as fertilizer (< 15 kg corm biomass per kg N). These results are in close agreement with data from previous studies [e.g (Goenaga and Chardon, 1995; Fa’amatuainu et al., 2016)] and together suggest that taro is physiologically sensitive to N fertilization. Either sub- or supra-optimal N rates can negatively affect corm yield and encourage vegetative growth (Osorio et al., 2003). Future research on taro should consider the timing of N application and split applications based on NUE, particularly during the first three to four months following crop establishment, given the sensitivity of the crop to N during this phase of development (Jacobs and Clarke, 1993; Goenaga, 1995; Beletse et al., 2025). Our research also indicates that increasing N supply has the potential to change the partitioning of dry matter and N content between parts of the plant in favor of above-ground components or cormels. Limiting N supply during corm initiation while maintaining an adequate and balanced supply of P and K, and then moderately increasing supply during corm filling could be an effective and economic way to limit the allocation of biomass to cormels and shoots while promoting the growth of a single corm.

Nitrogen is often the most limiting mineral nutrient for taro growth in farmer managed systems. Therefore, it is vital that any adaptations to crop management consider the potential effects of nitrogen on taro development. Management strategies for improving corm yield should consider the equivalent of 10 mM N in order to maximize total biomass production without compromising nutritional quality. However, such response to nitrogen is contingent on adequate supplies of both phosphorus and potassium. In this study, partitioning of biomass to the corms was reduced at high N levels (20 mM N) and plant growth was restricted, possibly due to nutrient imbalances. The timing of N application to taro crops may also be important. Here, lower rates of fertilizers during corm formation favored root development and limited the number of suckers. Higher rates were more beneficial during corm filling, but not beyond, when allocation reverted to the leaves. The dataset generated by this study will support the development of the taro module within the APSIM modelling framework, such as the one presented in Beletse et al. (2025), to improve management strategies that will optimize yields and promote adaptation of taro cropping systems to climate change. Further studies of the physiological responses of under studied crops, such as taro, to different environmental conditions are urgently needed to help improve, achieve and maintain food security. This is an important consideration for Pacific Island countries where food shortages are common as a result of recurrent climate-related disasters.

## 5 Conclusion

The results of this study indicate that nitrogen availability is a key determinant of biomass production, partitioning and nutritional composition in taro. Relative growth rate and main corm biomass were highest in plants supplied with 5 and 10 mM N, with some variation from harvest to harvest. Biomass accumulation and the number of cormels were limited by very low nitrogen (2.5 mM N). Growth was also suppressed when nitrogen supply was high (20 mM N), possibly due to nutrient imbalances. Given the high cost of fertilizers, we conclude that applying modest amounts of nitrogenous fertilizer (i.e. 10 mM N nutrient solution, or approximately 8–9 g per plant), is sufficient to promote the growth of the main corm. This work aids the development of management strategies for taro aimed at optimizing yields, improving nitrogen use efficiency and generating baseline data for further nutrient experiments.

## Data Availability

The data presented in this study can be found online at https://doi.org/10.26180/30423973 (Gleadow et al., 2025).
