# Supplementary material for: Optimising plant growth, biomass partitioning, and nitrogen use efficiency in taro (Colocasia esculenta (L.) Schott)
Source: Front Plant Sci. 2026 Jan 29;16:1731490. doi: 10.3389/fpls.2025.1731490 (PMC12894226; doi:10.3389/fpls.2025.1731490)
Supplement: Supplementary file 1 [file DataSheet1.pdf]

frontiers

Supplementary Material

Optimizing plant growth, biomass partitioning, and nitrogen use efficiency in taro (*Colocasia esculenta* (L.) Schott)

L. Steel, D.L. Antille, R.M. Gleadow*

*Corresponding author: ros.gleadow@monash.edu

Supplementary Tables

- Table S1: pH of soil and nutrient solutions.
- Table S2: Light intensity at different positions within the taro canopy and across the greenhouse.
- Table S3: Height, leaf number and biomass of taro plants at each harvest, and Relative Growth Rate calculated for the intervals between harvests.

Supplementary Table S1. Average soil pH for each of the five nitrogen treatments, taken after the final harvest, and pH of the nutrient solution (administered twice per week). Pots were flushed with water once per week to prevent salt build-up. Average soil pH is the mean of five replicates ± 1 SE. The N:P ratio of the nutrient solution was calculated from the components on a mole:mole basis.

N treatment	N:P ratio	Average soil pH	Nutrient solution pH
2.5 mM	1.5	6.13 ± 0.17	3.73
5 mM	8.5	5.88 ± 0.04	3.55
10 mM	17.0	5.80 ± 0.05	3.50
15 mM	25.5	5.38 ± 0.16	3.80
20 mM	34.0	5.24 ± 0.07	3.50

Supplementary Table S2. The average light intensity (µmol quanta m^{-2} s^{-1}) at different positions in the plant canopy (top, middle, and bottom) for plants of the five nitrogen treatment groups, at four positions within the glasshouse on 14 May 2015. Each mean is the average of four replicates ± 1 SE.

		Glasshouse Position (GP)			
**Treatment**	**Canopy level**	**GP1**	**GP2**	**GP3**	**GP4**
2.5 mM	Top	171.50 ± 9.41	324.00 ± 20.13	343.73 ± 8.34	471.05 ± 12.67
	Middle	100.55 ± 20.16	116.16 ± 16.51	195.35 ± 33.30	341.30 ± 74.50
	Base	61.56 ± 17.59	54.12 ± 4.32	78.34 ± 10.75	128.51 ± 16.82
5 mM	Top	170.23 ± 17.05	251.48 ± 27.53	321.13 ± 15.40	552.58 ± 30.18
	Middle	82.19 ± 7.65	110.69 ± 21.88	185.70 ± 42.56	323.40 ± 92.26
	Base	66.83 ± 10.28	68.82 ± 10.97	95.72 ± 15.87	173.13 ± 30.93
10 mM	Top	153.18 ± 26.56	223.85 ± 36.59	283.05 ± 12.61	631.65 ± 19.95
	Middle	67.77 ± 17.86	93.69 ± 18.10	183.73 ± 14.09	357.30 ± 93.06
	Base	50.78 ± 8.06	50.93 ± 16.77	59.85 ± 12.45	90.76 ± 15.97
15 mM	Top	229.55 ± 31.48	237.58 ± 20.30	350.38 ± 13.23	407.85 ± 23.88
	Middle	73.90 ± 7.99	119.64 ± 43.13	206.28 ± 25.71	255.93 ± 74.11
	Base	77.69 ± 19.74	72.35 ± 12/78	101.37 ± 14.45	158.85 ± 28.11
20 mM	Top	239.45 ± 25.23	188.55 ± 20.90	181.23 ± 21.17	191.55 ± 6.86
	Middle	96.50 ± 22.76	64.23 ± 19.00	74.83 ± 13.80	97.88 ± 20.23
	Base	121.44 ± 18.78	80.58 ± 14.71	61.31 ± 13.52	72.73 ± 20.00

Supplementary Table S3. Total biomass (dry weight in g), height (cm), and leaf number of taro plants grown in nutrient solutions containing five different concentrations of nitrogen (supplied as 6 NO_3; 1 NH_4) and harvested over a 9-month period.

This table is in four parts, one for each harvest date:

- *Table S3A. Harvest 1: 35 Days After Planting, approx. 2 weeks before treatments began.*
- *Table S3B. Harvest 2: 124-125 DAP approx. 2.5 months after treatments began.*
- *Table S3C: Harvest 3: 245-265 DAP, approx. 7 months after treatments began.*
- *Table S3D: Harvest 4: 298-305 DAP, approx. 8.5 months after treatments began.*

Supplementary Table 3A. Harvest 1. Total biomass (dry weight in g), height (cm), and leaf number of taro harvested 16 days before the nitrogen treatments began (35 DAP). Each mean is the average of 12 replicates ± 1 SE. Plants did not have any suckers. Leaf number is the average number of all green leaves (rolled, peeping, expanded, and expanding).

Parameter	Mean ± 1SE
**Total biomass (g dw)**	4.91 ± 0.52
- *Petioles*	0.88 ± 0.09
- *Leaf*	0.73 ± 0.09
- *Corm*	2.86 ± 0.50
- *Roots*	0.45 ± 0.11
**Height (cm)**	31.21 ± 1.32
**Leaf number**	3.25 ± 0.20

Supplementary Table 3B. Harvest 2. Biomass (dry weight in g) and growth of taro grown under five different levels of nitrogen for 2.5 months (124-125 DAP). Each mean is the average of 5-6 replicates ± 1 SE. RGR-H1 (relative growth rate, g g^{-1} day^{-1}) is calculated relative to Harvest 1. RGR was calculated for the interval between Harvest 1 and Harvest 2 (corm formation). Leaf number is the average number of all green leaves (rolled, peeping, and expanded, *sensu* Lloyd *et al.* 2021). Means in rows with the same superscript are not significantly different at $p < 0.05$ using post-hoc Tukey tests.

	**2.5mM**	**5mM**	**10mM**	**15mM**	**20mM**	**p-value**
**Biomass (g dw)**						
Total	20.70 ± 2.93	24.52 ± 8.69	27.11 ± 5.93	28.85 ± 2.86	17.20 ± 2.44	0.510
- *Leaf*	2.53 ± 0.35^{A}	3.00 ± 0.78^{AB}	3.44 ± 0.77^{AB}	5.09 ± 0.59^{B}	2.81 ± 0.49^{AB}	0.053
- *Petioles*	4.04 ± 0.76	4.85 ± 1.23	4.94 ± 1.11	6.98 ± 0.50	4.75 ± 0.77	0.244
- *Corm*	1.97 ± 0.57	5.46 ± 4.50	6.47 ± 4.86	2.39 ± 0.80	1.67 ± 0.50	0.706
- *Roots*	12.17 ± 2.22	10.23 ± 2.73	11.20 ± 1.76	11.65 ± 2.61	7.92 ± 1.67	0.692
- *Suckers*	0.00	0.99 ± 0.99	1.05 ± 0.80	2.75 ± 2.32	0.06 ± 0.04	0.489
**RGR-H1**	0.0157 ± 0.0013	0.0144 ± 0.0041	0.0181 ± 0.0021	0.0188 ± 0.0013	0.0134 ± 0.0022	0.359
**Height (cm)**	49.08 ± 3.10	52.92 ± 3.83	53.00 ± 2.49	56.83 ± 2.22	55.33 ± 2.19	0.390
**Sucker number**	0.00	0.50 ± 0.50	0.33 ± 0.33	0.50 ± 0.34	0.17 ± 0.17	0.759
**Leaf number**	3.17 ± 0.17^{A}	3.17 ± 0.31^{A}	3.83 ± 0.31^{AB}	4.17 ± 0.31^{B}	3.17 ± 0.17^{A}	0.027

Supplementary Table 3C. Harvest 3. Biomass (dry weight in g) and growth of taro grown under five different levels of nitrogen for 7 months (245-265 DAP). Each mean is the average of six replicates ± 1 SE. RGR (relative growth rate, g g^{-1} day^{-1}) is calculated relative to Harvest 1. Leaf number is the average number of all green leaves (rolled, peeping, and expanded). Means in rows with the same superscript are not significantly different at $p < 0.05$ using post-hoc Tukey tests.

	2.5 mM	5 mM	10 mM	15 mM	20 mM	p-value
**Biomass (g dw)**						
Total	49.72 ± 7.72^{A}	113.17 ± 5.18^{B}	115.89 ± 24.38^{B}	60.91 ± 4.20^{A}	39.32 ± 10.64^{A}	0.0003
- *Leaf*	3.30 ± 0.19^{A}	4.57 ± 0.77^{AB}	7.72 ± 1.60^{B}	2.73 ± 0.95^{A}	2.56 ± 1.01^{A}	0.0080
- *Petioles*	2.77 ± 0.52	2.95 ± 0.79	5.17± 1.55	2.09 ± 0.51	2.58 ± 0.94	0.2020
- *Corm*	28.54 ± 4.63^{A}	61.07 ± 7.40^{B}	60.70 ± 19.75^{B}	28.23 ± 2.72^{A}	19.79 ± 4.53^{A}	0.0133
- *Roots*	2.93 ± 0.35^{A}	2.52 ± 0.51^{A}	0.83 ± 0.30^{B}	0.37 ± 0.10^{B}	0.50 ± 0.19^{B}	<0.0001
- *Suckers*	12.18 ± 4.05^{A}	42.06 ± 9.48^{B}	41.47 ± 6.42^{B}	27.49 ± 1.44^{AB}	13.83 ±7.07^{A}	0.0035
**RGR**	0.0103 ± 0.0007^{A}	0.0142 ± 0.0002^{B}	0.0138 ± 0.0010^{B}	0.0107 ± 0.0003^{AB}	0.0086 ± 0.0013^{A}	0.0001
**Height (cm)**	52.50 ± 1.93	51.92 ± 4.78	58.25 ± 4.81	47.50 ± 2.99	44.33 ±5.32	0.2020
**Sucker number**	2.67 ± 0.99^{A}	5.00 ± 0.73^{AB}	7.33 ± 0.76^{B}	5.50 ± 0.56^{A}	3.50 ± 1.23^{A}	0.0097
**Leaf number**	2.33 ± 0.33	2.17 ± 0.31	4.00 ± 1.46	2.33 ± 0.61	1.67 ± 0.33	0.271

Supplementary Table 3D. Harvest 4. Biomass and growth of taro grown under five different levels of nitrogen for 8.5 months (298-305 DAP). Each mean is the average of 6-9 replicates ± 1 SE. RGR (relative growth rate, g g^{-1} day^{-1}) is calculated relative to Harvest 1. Leaf number is the average number of all green leaves (rolled, peeping, and expanded). Means in rows with the same superscript are not significantly different at $p < 0.05$ using post-hoc Tukey tests.

	2.5 mM	5 mM	10 mM	15 mM	20 mM	p-value
**Biomass (g dw)**						
Total	74.77 ± 10.12^{A}	147.32 ± 10.93^{BC}	168.72 ± 13.78^{C}	102.91 ± 11.03^{AB}	66.49 ± 12.58^{A}	<0.0001
- *Leaf*	3.071± 0.54^{A}	4.49 ± 0.59^{A}	3.67 ± 0.96^{A}	4.78 ± 0.93^{A}	1.32 ± 0.4^{B}	0.040
- *Petioles*	5.10 ± 0.75	5.24 ± 0.84	4.88 ± 1.34	5.45 ± 1.07	1.61 ± 0.42	0.107
- *Corm*	45.91 ± 7.06^{A}	90.20 ± 7.54^{B}	77.10 ± 8.69^{B}	44.79 ± 5.24^{A}	36.20 ± 8.11^{A}	<0.0001
- *Roots*	3.12 ± 0.52^{A}	2.18 ± 0.27^{A}	0.65 ± 0.11^{B}	0.54 ± 0.15^{B}	0.21 ± 0.06^{B}	<0.0001
- *Suckers*	15.16 ± 4.60^{A}	49.75 ± 5.24^{AB}	82.42 ± 6.89^{C}	47.35 ± 9.36^{B}	34.65 ± 10.30^{A}	<0.0001
**RGR**	0.0114 ± 0.0005^{A}	0.0127 ± 0.0006^{B}	0.0132 ± 0.0003^{B}	0.0113 ± 0.0008^{A}	0.0094 ± 0.0007^{A}	<0.0001
**Height (cm)**	58.44 ± 1.88	61.56 ± 2.60	51.06 ± 6.33	56.75 ± 3.88	44.08 ± 3.91	0.067
**Sucker number**	2.67 ± 0.62^{A}	6.13 ± 0.44^{BC}	8.00 ± 0.53^{C}	5.88 ± 0.77^{BC}	5.00 ± 0.68^{AB}	0.016
**Leaf number**	2.33 ± 0.67	1.75 ± 0.45	2.33 ± 0.65	2.25 ± 0.49	2.50 ± 0.62	0.926
